# Is Seeing Cigarettes in the Retail Environment Associated With Impulse Purchases? Findings From Surveys in Disadvantaged and Non-disadvantaged Neighborhoods in the Netherlands

**DOI:** 10.1093/ntr/ntaf026

**Published:** 2025-02-07

**Authors:** Nikita L Poole, Floor A van den Brand, Marc C Willemsen, Gera E Nagelhout

**Affiliations:** Department of Health Promotion, Care and Public Health Research Institute (CAPHRI), Maastricht University, Maastricht, the Netherlands; Research Institute IVO, The Hague, the Netherlands; Department of Family Medicine, Care and Public Health Research Institute (CAPHRI), Maastricht University, Maastricht, the Netherlands; Department of Health Promotion, Care and Public Health Research Institute (CAPHRI), Maastricht University, Maastricht, the Netherlands; The Netherlands Expertise Center for Tobacco Control (NET), Trimbos Institute, Utrecht, the Netherlands; Department of Health Promotion, Care and Public Health Research Institute (CAPHRI), Maastricht University, Maastricht, the Netherlands; Centre of Expertise Perspective in Health, Avans University of Applied Sciences, Breda, the Netherlands

## Abstract

**Background:**

The retail environment plays an important role in impulse purchase behavior. This study aims to examine the extent to which different sources of exposure to tobacco in the Dutch retail environment are associated with impulse purchases of tobacco. We investigate whether this association differs based on neighborhood disadvantage, demographic and smoking characteristics.

**Aims and Methods:**

We employed a cross-sectional online survey design among 1223 Dutch adults who smoke from a probability-based panel database, with half residing in a disadvantaged neighborhood. We conducted multiple logistic regression analyses for impulse purchases with six sources of reported tobacco exposure in the retail environment. We tested for interactions with neighborhood disadvantage, demographic and smoking characteristics.

**Results:**

Five sources of exposure were associated with impulse purchases: seeing cigarette packages at the counter or checkout, tobacco advertisements, people smoking by the shop entrance, a friend buying cigarettes and a family member buying cigarettes. We found one significant interaction in the relationship between exposure and impulse purchases by previous quit attempts. Individuals who have attempted or intend to quit, younger respondents, and frequent tobacco purchasers were more likely to be exposed to tobacco and make impulse purchases. Living in a disadvantaged neighborhood was associated with greater exposure to tobacco in the retail environment.

**Conclusions:**

Several sources of exposure to tobacco in the retail environment, most notably seeing cigarettes at the checkout and family buying cigarettes, are associated with impulse tobacco purchases. These findings provide support for limiting the sale of tobacco to specialist shops to prevent impulse purchases.

**Implications:**

This study shows that even with a point-of-sale display ban, several other types of exposure to tobacco in the retail environment may trigger adults who smoke to make an impulse purchase. We provide further evidence that certain groups are at an increased risk of being exposed to tobacco and making impulse purchases. This study provides support for limiting the sale of tobacco to specialist shops. These findings are directly relevant not only for the Netherlands, but for other nations considering their next steps for tobacco control.

## Introduction

Tobacco impulse purchases are often triggered by environmental and situational cues, such as seeing tobacco point-of-sale (POS) promotional displays, drinking alcohol or being around people who are smoking or purchasing tobacco.^1–3^ Studies indicate that around 11%–30% of people who smoke report making impulse purchases at least sometimes,^1,4,5^ with 28.3% of those reporting making unplanned purchases doing so once per week in one study.^1^ Minimizing tobacco impulse purchases is particularly important for people trying to quit smoking to prevent relapse.^1^

The visibility of tobacco in the retail environment is a key area of study when examining impulse purchases, as visibility of tobacco can trigger emotional and physical responses, increasing temptation to buy.^6^ Policies to reduce the visibility of tobacco product displays have been implemented in 50 countries as of 2020 (World Health Organization, 2022) and their effects have been evaluated, highlighting the importance of the retail environment in smoking behavior, tobacco exposure, and purchasing behavior.^2,7–9^ With POS display bans being increasingly implemented, it is important to understand whether and how people may otherwise be triggered to make an impulse purchase in the retail environment. For instance, evidence suggests that merely seeing a place where tobacco is sold may trigger thoughts and urges to purchase tobacco.^10^ Standing at a checkout where cigarettes are available to purchase and seeing others purchase tobacco are also reasons given for unplanned purchases.^1,3^

Although more research is needed to confirm these findings, studies indicate that certain individuals may be more susceptible to making impulse purchases. Research so far has found that those who are more susceptible to making impulse purchases tend to notice POS displays more often, are more often single,^11^ female,^4^ younger, have a lower level of education,^12^ smoke less frequently, have recently made a quit attempt,^3,11^ and intend to quit in the upcoming months.^3,4,11^ Previous research has also found that those who visit shops daily and those who are younger are more likely to notice cigarette pack displays^4^ and that younger adults are also more vulnerable to tobacco marketing.^13^ We hypothesize that impulse purchasing is higher among these groups as they may be more heavily influenced by exposure to tobacco, which in turn may increase the likelihood of them making an impulse purchase.

Whilst the relationship between the socioeconomic status of a neighborhood and impulse purchases has to our knowledge not yet been studied, there is evidence that exposure to tobacco and tobacco promotion in the retail environment is greater in neighborhoods with a lower socioeconomic status,^14^ which may go on to trigger impulse purchasing much in the way that POS have been found to.^2^ For instance, several studies have illustrated that a higher density of tobacco POS is found in disadvantaged neighborhoods,^15–17^ which in turn is associated with income and ethnicity disparities in tobacco use,^18^ and a decreased likelihood of smoking cessation.^19,20^ In addition, exposure to tobacco may be higher in disadvantaged neighborhoods due to lower compliance to in-shop regulations.^21^

In the Netherlands, POS displays have been banned since 1 July 2020 in supermarkets, followed by 1 January 2021 for other POS, with the exception of tobacco specialty shops.^22^ This measure, along with measures to limit the sale of tobacco in supermarkets and the hospitality industry (implemented 1 July 2024), is part of the Dutch National Prevention Agreement which has the ambition to achieve a smoke-free generation (less than 5% adult smoking prevalence) by 2040.^23^ The Dutch government has further outlined its intention to limit the sale of tobacco to specialty tobacco shops by 2032. The objective of limiting the places where tobacco can be sold is to reduce the availability of tobacco and the possibility for young people to be exposed to tobacco in most shop environments^24^ and to reduce the temptation to smoke among people who are trying to quit.

This study aims to investigate the extent to which exposure to tobacco in the retail environment is associated with impulse purchases. Secondly, we are interested in whether exposure to tobacco in the retail environment, impulse purchases, and the relationship between the two differs based on demographic and smoking characteristics, and neighborhood disadvantage.

## Methods

### Design and Setting

Our study employs a cross-sectional online survey design. Data was collected between 26 and 29 October 2022. We used data collected by a large probability-based panel database (NIPObase, conducted by Verian). Participants were contacted to participate via e-mail, and received points from NIPObase for questions they answered, which could be exchanged for a gift certificate or a donation to charity. Further information on the study design and sampling are described in more detail elsewhere.^25^

### Sample

Our sample consisted of Dutch adults who smoke (factory made or roll-your own) cigarettes. Eligible participants smoked at least monthly, have smoked at least 100 cigarettes in their lifetime and were aged 18 years or older. This resulted in a sample of 1233 respondents. All participants lived in the Netherlands, with half living in a disadvantaged neighborhood (*n* = 613) and the other half living in a non-disadvantaged neighborhood (*n* = 610). To distinguish between disadvantaged and non-disadvantaged neighborhoods, we made use of the Dutch Liveability measure (*Leefbarometer*)^26,27^ and the Socioeconomic scores of private households (SES-WOA)^28^ measure from Statistics Netherlands. Neighborhoods with a score at the postcode level that fell within the lowest 4 of 9 categories of the liveability measure (rated “very unsatisfactory” (1) to “weak” (4)) and/or the lowest 20% of scores from the SES-WOA were classified as a disadvantaged neighborhood.^25^

### Measures

#### Impulse Purchases

The primary outcome variable was cigarette impulse purchases. This was measured with the following question: “*An impulse purchase is an unplanned purchase of cigarettes. You are in a shop to buy something other than cigarettes. How often do you decide to buy cigarettes as an impulse purchase?*.*”*^12^ Responses ranged on a 5-point scale from “Never” (1) to “Always” (5) and were later dichotomized into “never” and “ever” making an impulse purchase. Respondents were also able to answer “Don’t know/don’t want to say,” which accounted for 0.7% of all responses. This was treated as missing for the analyses.

#### Perceived Tobacco Exposure in the Retail Environment

Six sources of exposure to tobacco in the retail environment were measured. We asked “*How often have you seen the following when visiting a shop in the last month*” for the following sources of exposure: (1) cigarette packages at the counter or checkout; (2) smoking accessories (for example lighters, filters, rolling paper); (3) advertisements for tobacco; (4) people smoking at the entrance of the shop; (5) a friend buying cigarettes; (6) a family member buying cigarettes. Respondents could answer on a 5-point scale from “Never” (1) to “Very often” (5) to indicate the extent to which they perceived to have been exposed. Respondents were also able to answer “Don’t know/don’t want to say,” accounting for 2.2%, 3.4%, 6.7%, 2.1%, 2.5% and 2.4% of responses per respective variable. This was recoded as “Never” for analyses.

#### Control Variables

*Demographic Characteristics*. Participants were recorded as being either a man or a woman (other sex/gender options were not given in the standard questionnaire of Verian) and the respondents’ age was reported in years. Age was categorized into 4 groups: 18–34; 35–49; 50–64; 65+.

Educational level was obtained by asking “*What is the highest level of education you have completed?*.” Responses were categorized into 3 groups: low (primary education and lower pre-vocational secondary education); moderate (middle pre-vocational secondary education and secondary vocational education); and high (senior general secondary education, (pre-) university education and higher professional education). Respondents were also able to answer “Don’t know/don’t want to say” (0.4% of all responses).

Gross monthly household income was measured with response options ranging on a 13-point scale from “Less than 750 euros per month” (1) to “More than 7000 euros per month” (13). Respondents were also able to answer “Don’t know/don’t want to say.” Answers were categorized into tertiles: ≤€2500/month; €2500/month to €4000/month; ≥€4000/month, in addition to the option “Don’t know/don’t want to say” (21.6% of all responses).

Smoking characteristics. The average number of cigarettes smoked per day was measured with “*On the days that you smoke, how many manufactured or roll-your-own cigarettes do you smoke on average per day?*” Respondents could indicate a number from 0 to 100 via a drop-down menu, or “Don’t know/don’t want to say” (1.5% of all responses). Those who indicated smoking weekly and monthly were also asked how many days per week/month they smoke, in order to calculate an average number of cigarettes per day. These were categorized into 4 groups: 0–5 cigarettes per day, 6–10 cigarettes per day, 11–20 cigarettes per day, and 21+ cigarettes per day.

Intention to quit was measured by asking “*Are you planning to quit smoking cigarettes within the next 6 months?*” Response options ranged on a 5-point scale from “Very unlikely” (1) to “Very likely” (5) and “Don’t know/don’t want to say.” The last option (accounting for 3.7% of all responses) was coded as the mid-point of the Likert scale (“Maybe, maybe not”) for the analyses.^29^

To measure previous quit attempts, participants were asked “*How many serious quit attempts (longer than 24 hours) have you made in the past 12 months?*” Answers on a scale of 0–100 were possible. Respondents were also able to answer “Don’t know/don’t want to say” (accounting for 1.5% of all responses) which was treated as zero quit attempts made. These responses were later dichotomised as “Yes” or “No” to a quit attempt being made in the past 12 months.

Frequency of tobacco purchase was measured by asking “*How often do you typically buy cigarettes or roll-your-own tobacco?*” with responses ranging on a 7-point scale from (1) “Nearly every day,” (2) “2-3 times per week,” (3) “Once per week,” (4) “2-3 times per month,” (5) “Once per month,” (6) “Less than once per month,” (7) “Never” and “Don’t know/don’t want to say” (0.4% of all responses).

Unless otherwise mentioned, the “Don’t know/don’t want to say” answer responses were treated as missing data. This procedure resulted in 2.7% missing data.

### Ethics

This study received ethical approval by the Research Ethics Committee of the Faculty of Health, Medicine and Life Sciences at Maastricht University (FHML-REC/2022/081). Respondents completed an online informed consent before taking part.

### Analyses

All analyses were performed in SPSS 26. We first tested for differences in impulse purchases and mean exposure to tobacco in the retail environment for the following characteristics: gender, age, educational level, income level, neighborhood disadvantage, average number of cigarettes per day, frequency of tobacco purchase, past quit attempts and intention to quit. Given the binary measurement of impulse purchases, we tested for differences between groups by means of chi-square tests. Differences in mean exposure to tobacco in the retail environment were tested with t-tests (for binary independent variables gender, neighborhood disadvantage and previous quit attempts) and one-way ANOVAs (for age, education, income, average number of cigarettes per day, intention to quit smoking, and frequency of tobacco purchase). Post hoc associations were examined by means of Tukey HSD.

Subsequently, we conducted multiple logistic regression analyses for the outcome impulse purchases, with the six different sources of tobacco exposure in the retail environment as the independent variables (seeing cigarette packages at the counter or checkout; seeing smoking accessories; seeing people smoking at the entrance of the shop; seeing a friend buying cigarettes; seeing a family member buying cigarettes). Our initial models tested the relationship between impulse purchase and the independent exposure variables separately from each other. Never making an impulse purchase was the reference category.

Secondly, we examined the relationship between impulse purchase and all exposures taken together, as there may be some degree of overlap in the exposure sources (eg one may see cigarettes packages at the checkout while a friend is buying cigarettes). This was done with 2 models. In the first of the 2 models, we controlled for gender, age, educational level, income level, average number of cigarettes per day, making a quit attempt in the past 12 months and intention to quit. In the second of the 2 models, frequency of tobacco purchase was added as a control variable, as this may be associated with the average number of cigarettes per day, impulse purchasing and tobacco exposure. We tested for multicollinearity between the independent variables. No multicollinearity was found. Two predictors, seeing someone smoking at the entrance of the shop and seeing a family member buying cigarettes, were found to have a nonlinear relationship with impulse purchases. These variables were therefore included in the analysis as 4 dummy variables.

Additional analyses were conducted in which only respondents who reported to never buy their cigarettes in tobacco specialty shops were included (*n* = 601), as these shops are exempt from the national POS display ban. In crosstabs analysis of the exposure variables with impulse purchasing, three exposure variables (seeing advertisements, seeing a friend buying cigarettes, seeing family member buying cigarettes) were found to have a count fewer than 5 in at least 1 cell and were thus collapsed from 5 to 3 categories: very often and often, sometimes and rarely, and never.

Lastly, to test for interactions between exposure and demographic and smoking characteristics on their effect on impulse purchases of tobacco, we used an average of the six variables for perceived tobacco exposure in the retail environment. Characteristics tested for interaction between exposure and impulse purchases of tobacco were demographic characteristics (gender, age, educational level), smoking characteristics (average number of cigarettes per day, frequency of tobacco purchase, making a quit attempt in the past 12 months and intention to quit), and neighborhood disadvantage. These interaction terms were added to the main model. Results with a *p*-value of <.05 were deemed significant.

## Results

### Response and Sample

Of the 2799 panel members invited, 10.4% (*n* = 292) were deemed ineligible to participate at screening and a further 7% (*n* = 196) did not complete the survey. The survey was completed by 43.7% of those invited (*n* = 1223).

The largest demographic groups in the included sample were aged 50–64 (35.2%), moderately educated (44.5%) with a low income level (28.4%). There were almost as many men (50.4%) as women in the sample. Participants most frequently reported smoking 11–20 cigarettes per day (39.9%) (Table 1).

**Table 1.** Demographic characteristics of the sample (*n* = 1223 unless otherwise stated)

	*n* (%)
**Gender**	
Male	616 (50.4)
Female	607 (49.6)
**Age**	
18–34	138 (11.3)
35–49	319 (26.1)
50–64	431 (35.2)
65+	335 (27.4)
**Education (*n* = 1218)**	
Low	390 (31.9)
Moderate	544 (44.5)
High	284 (23.2)
**Income**	
Low	347 (28.4)
Moderate	333 (27.2)
High	279 (22.8)
Don’t know/Don’t want to say	264 (21.6)
**Average number of cigarettes per day (*n* = 1205)**	
0–5	271 (22.6)
6–10	327 (27.3)
11–20	479 (39.9)
21+	123 (10.3)

Supplementary Tables 1 and 2 present the frequencies with which respondents reported seeing each source of tobacco exposure. Supplementary Table 1 presents frequencies for the whole sample, while Supplementary Table 2 presents the frequencies of exposure for only those who do not buy cigarettes at speciality tobacco shops (*n* = 601). In the main sample, respondents most frequently reported seeing smoking accessories (for example lighters, filters, rolling paper) (21.7% often) and someone smoking at the shop entrance (19.0% often).

### Differences in perceived exposure and impulse purchases to tobacco in the retail environment

Differences in impulse purchases were found based on age ($\chi^{2}$=48.580, *p* < .001), intention to quit ($\chi^{2}$=70.671, *p* < .001) and frequency of tobacco purchase ($\chi^{2}$=47.371, *p* < .001). Post hoc tests using Bonferroni-adjusted alphas found that among those aged 18–34 were more likely to make impulse purchases than never making them ($\chi^{2}$=39.690, *p* < .001) and those aged 65 and over were more likely to report never making impulse purchases ($\chi^{2}$=17.640, *p* < .001). Regarding intention to quit, those who said that they were likely to quit in the next 6 months reported making impulse purchases the most out of any other answer category (47.9%). However post hoc analyses showed that they were still more likely to never impulse purchase ($\chi^{2}$=13.690, *p* < .001), as were those who were very likely to quit in the next 6 months ($\chi^{2}$=21.160, *p* = .002). Lastly, those who reported buying tobacco nearly every day were more likely to report making impulse purchases than never making them ($\chi^{2}$=18.490, *p* < .001), while those who buy 2–3 times per week and less than once per month were more likely to never to make impulse purchases ($\chi^{2}$=16.810, *p* < .001; $\chi^{2}$=10.890, *p* < .001). Those who reported making a quit attempt in the past 12 months were more likely to make an impulse purchase ($\chi^{2}$=51.085, *p* < .001) (Table 2).

**Table 2.** Difference in impulse purchases and mean exposure to tobacco in the retail environment based on demographic and smoking characteristics

	Impulse purchase *n* (% yes)	*p*-value	Exposure to tobacco mean† (SD)	*p*-value
**Gender**				
Man	211 (34.5)	$\chi^{2}$ =0.001	2.10 (0.72)	*t* = 0.885
Woman	208 (34.4)	*p* = .972	2.06 (0.70)	*p* = .377
**Age**				
18–34	80 (58.8)	$\chi^{2}$ =48.580	2.35 (0.84)	*F*(3,1219) = 12.779
35–49	109 (34.5)	***p* < .001**	2.11 (0.68)	***p* < .001**
50–64	146 (34.0)		2.09 (0.72)	
65+	84 (25.1)		1.92 (0.64)	
**Education**				
Low	122 (31.6)	$\chi^{2}$ =4.895	2.05 (0.75)	*F*(2,1215) = 0.381
Moderate	184 (33.9)	*p* = .087	2.09 (0.70)	*p* = .683
High	112 (39.7)		2.09 (0.67)	
**Income**				
Low	127 (36.8)	$\chi^{2}$ =2.790	2.14 (0.75)	*F*(3,1219) = 1.274
Moderate	118 (35.4)	*p* = .425	2.07 (0.68)	*p* = .282
High	95 (34.2)		2.04 (0.66)	
Don’t know/Don’t want to say	79 (30.5)		2.06 (0.75)	
**Neighborhood disadvantage**				
Yes	222 (36.3)	$\chi^{2}$ =1.858	2.14 (0.73)	*t* = −3.075
No	197 (32.6)	*p* = .173	2.02 (0.68)	***p* = .002**
**Average number of cigarettes per day**				
0-5	159 (59.1)	$\chi^{2}$ =7.456	2.04 (0.68)	*F*(3,1196) = 0.617
6–10	212 (65.2)	*p* = .059	2.11 (0.69)	*p* = .604
11–21	324 (67.9)		2.07 (0.72)	
21+	86 (70.5)		2.11 (0.71)	
**Past quit attempts**				
Yes	254 (47.4)	$\chi^{2}$ =51.085	2.17 (0.76)	*t* = -3.167
No	165 (24.3)	***p* < .001**	2.01 (0.67)	***p* < .001**
**Intention to quit smoking**				
Very likely	82 (42.7)	$\chi^{2}$ =38.332	2.05 (0.72)	*F*(4,1218) = 3.997
Likely	104 (47.9)	***p* < .001**	2.24 (0.77)	***p* = .003**
Maybe, maybe not	138 (31.7)		2.04 (0.68)	
Unlikely	43 (28.3)		1.97 (0.62)	
Very unlikely	52 (23.7)		2.09 (0.74)	
**Frequency of tobacco purchase**				
Nearly every day	28 (65.1)	$\chi^{2}$ =47.371	2.52 (1.01)	*F*(6,1211) = 4.336
2–3 times per week	140 (43.9)	***p* < .001**	2.15 (0.68)	***p* < .001**
Once per week	129 (30.7)		2.04 (0.69)	
2–3 times per month	46 (30.9)		2.05 (0.72)	
Once per month	55 (33.3)		2.02 (0.71)	
Less than once per month	18 (18.9)		1.96 (0.63)	
Never	3 (15.0)		2.18 (0.77)	

† Exposure to tobacco mean: an average score of 6 exposure sources, rated 1 ‘*Never’* to 5 *“Very often”:* cigarette packages at the counter or checkout; smoking accessories; advertisements for tobacco; people smoking at the entrance of the shop; a friend buying cigarettes; a family member buying cigarettes. Significant results (*p* < .05) are indicated in bold.

Differences in mean exposure to tobacco were found based on age, intention to quit and frequency of tobacco purchase. Post hoc analyses revealed that those who were aged 18–24 were more likely to be exposed to tobacco than all other age groups (vs. 35–49 *p* = .007; vs. 50 + 64 *p* < .001; vs. 65 + *p* < .001). Those aged 35–49 and 50–64 were also more likely to be exposed to tobacco than those aged 65+ (*p* = .002 and *p* = .006, respectively). Those who were likely to quit in the next 6 months were more likely to be exposed to tobacco than those who were unlikely (*p* = .003) or neutral about quitting (*p* = .009). Lastly, those who buy tobacco products nearly every day were more likely to be exposed to tobacco than all other categories except “never” (vs. 2–3 times a week *p* = .018; vs. once per week *p* < .001; vs. 2–3 times per month *p* = .002; vs. once per month *p* < .001; vs. less than once per month *p* < .001) Those who reported making a quit attempt in the past 12 months and those living in a disadvantaged neighborhood also reported greater exposure (*t* = −3.167, *p* < .01 and *t* = −3.075, *p* = .001, respectively) (Table 2).

### Association between perceived exposure to tobacco in the retail environment and impulse purchases

We first tested for the relationship between impulse purchases and the 6 sources of exposure to tobacco in the retail environment separately from each other. Five of 6 exposure sources were associated with impulse purchases: seeing cigarette packages at the counter or checkout (OR = 1.28, 95%CI = 1.14–1.42, *p* < .001), seeing advertisements for tobacco (OR = 1.33, 95%CI = 1.15–1.54, *p* < .001), seeing people smoking at the entrance of the shop “sometimes” (OR = 1.61, 95%CI = 1.12–1.45, *p* = .010) and “often” (OR = 1.54, 95%CI = 1.02–2.34, *p* = .041), and seeing a friend (OR = 1.29, 95%CI = 1.14–1.56, *p* < .001) or family member buy cigarettes “rarely” (OR = 2.22, 95%CI = 1.55–3.18, *p* < .001), “sometimes” (OR = 2.11, 95%CI = 1.50–2.97, *p* < .001) and “often” (OR = 2.58, 95%CI = 1.55–4.30, *p* < .001) compared to “never” (Table 3).

**Table 3.** Logistic regression models of exposure to tobacco in the retail environment and impulse purchases

	Impulse purchases OR (95% CI)	*p*-value
**Sources of exposure separately** ^1^		
Cigarettes at the checkout	1.28 (1.14–1.42)	**<.001**
Smoking accessories (e.g. filters and lighters)	1.09 (0.98–1.29)	.100
Advertisements for tobacco	1.33 (1.15–1.54)	**<.001**
People smoking at the entrance of the shop		
Rarely	0.95 (0.62–1.45)	.749
Sometimes	1.61 (1.12–2.32)	**.010**
Often	1.54 (1.02–2.34)	**.041**
Very Often	1.48 (0.84–2.61)	.176
Friends buying cigarettes	1.29 (1.14–1.45)	**<.001**
Family buying cigarettes		
Rarely	2.22 (1.55–3.18)	**<.001**
Sometimes	2.11 (1.50–2.97)	**<.001**
Often	2.58 (1.55–4.30)	**<.001**
Very Often	1.01 (0.45–2.68)	.842
**Model 1** ^1^		
Cigarettes at the checkout	1.22 (1.07–1.38)	**.003**
Smoking accessories	0.96 (0.85–1.07)	.450
Advertisements for tobacco	1.11 (0.94–1.31)	.239
People smoking at the entrance of the shop		
Rarely	0.73 (0.47–1.14)	.168
Sometimes	1.24 (0.84–1.83)	.278
Often	1.02 (0.64–1.61)	.940
Very Often	0.99 (0.52–1.88)	.965
Friends buying cigarettes	1.10 (0.94–1.28)	.246
Family buying cigarettes		
Rarely	1.96 (1.34–2.87)	**<.001**
Sometimes	1.66 (1.13–2.43)	**.010**
Often	1.88 (1.06–3.33)	**.030**
Very Often	0.58 (0.21–1.63)	.305
**Model 2** ^2^		
Cigarettes at the checkout	1.24 (1.09–1.41)	**.002**
Smoking accessories	0.91 (0.81–1.03)	.132
Advertisements for tobacco	1.11 (0.94–1.33)	.223
People smoking at the entrance of the shop		
Rarely	0.76 (0.48–1.20)	.240
Sometimes	1.33 (0.89–1.99)	.161
Often	1.10 (0.69–1.77)	.693
Very Often	1.04 (0.54–2.02)	.901
Friends buying cigarettes	1.09 (0.93–1.28)	.286
Family buying cigarettes		
Rarely	1.88 (1.27–2.79)	**.002**
Sometimes	1.61 (1.09–2.38)	**.017**
Often	1.70 (0.95–3.05)	.074
Very often	0.51 (0.17–1.52)	.225

Significant results (*p* < .05) are indicated in bold.

In Models 1 and 2, all sources of exposure to tobacco in the retail environment were controlled for.

^1^Analyses controlled for gender, age, educational level, income level, average number of cigarettes per day, making a quit attempt in the past 12 months and intention to quit.

^2^Analyses controlled for gender, age, educational level, income level, average number of cigarettes per day, making a quit attempt in the past 12 months, intention to quit and frequency of tobacco purchase.

We subsequently examined whether these associations remained statistically significant when controlling for the other sources of exposure (Table 3). In the first model, statistically significant associations were seeing cigarettes at the counter or checkout (OR = 1.22, 95%CI = 1.07–1.38, *p* = .003) and seeing a family member buying cigarettes “rarely” (OR = 1.96, 95%CI = 1.34–2.87, *p* < .001), “sometimes” (1.66, 95%CI = 1.13–2.43, *p* = .010) and “often” (OR = 1.88, 95%CI = 1.06–3.33, *p* = .030) compared to “never.” In the second model, frequency of tobacco purchases was added to the control variables. The same predictors remained significantly associated with impulse purchases, with the exception of seeing a family member purchase cigarettes often, compared to “never” (Table 3).

In additional analyses among respondents who reported to never buy their cigarettes in tobacco specialty shops (not shown in tables), two sources of exposure were significantly associated with impulse purchases when analyzed separately: seeing advertisements for tobacco (OR = 1.59, 95%CI = 1.11–2.328, *p* = .012) and seeing friends buying cigarettes (OR = 1.52, 95%CI = 1.11–2.09, *p* = .010). When taking all sources of exposure together, associations presented in the same direction, however, no exposure variables were significantly associated with impulse purchases in both models (with and without frequency of tobacco purchases).

### Interactions with demographic, neighborhood, and smoking characteristics

Interactions of neighborhood deprivation, demographic, and smoking characteristics with mean exposure to tobacco in the retail environment revealed only one significant interaction (Table 4). The interaction between mean exposure and reporting making a quit attempt in the past 12 months was significant (OR = 1.56, 95%CI = 1.06–2.42, *p* = .047).

**Table 4.** Logistic regression models of the interaction terms of mean exposure to tobacco in the retail environment with demographic and smoking characteristics on impulse purchase of tobacco

	Impulse purchases OR (95% CI)	*p*-value
**Interactions with mean exposure** †		
Mean exposure * gender	0.79 (0.54–1.15)	.219
Mean exposure * age	0.99 (0.81–1.21)	.918
Mean exposure * education	1.17 (0.93–1.55)	.172
Mean exposure * income	0.91 (0.77–1.08)	.284
Mean exposure * neighborhood disadvantage	1.06 (0.72–1.55)	.772
Mean exposure * average number of cigarettes per day	0.99 (0.81–1.21)	.912
Mean exposure * past quit attempts	1.56 (1.06–2.42)	**.047**
Mean exposure * intention to quit smoking	1.01 (0.87–1.17)	.944
Mean exposure * frequency of tobacco purchase	1.07 (0.93–1.23)	.358

Significant results (*p* < .05) are indicated in bold.

† Exposure to tobacco mean: an average score of 6 exposure sources, rated 1 ‘*Never’* to 5 *“Very often”:* cigarette packages at the counter or checkout; smoking accessories; advertisements for tobacco; people smoking at the entrance of the shop; a friend buying cigarettes; a family member buying cigarettes.

Analyses controlled for main effects, gender, age, educational level, income level, average number of cigarettes per day, making a quit attempt in the past 12 months, intention to quit and frequency of tobacco purchase.

### Sensitivity Analysis

A sensitivity analysis in which the answer option “don’t know/don’t want to say” was coded as missing for the variables intention to quit, previous quit attempts and the six exposure variables was also conducted. Because the proportion of answers in this category for income (21.6% of all answers), this was kept as a fourth category. In these analyses, the exposure variables seeing friends and family purchase tobacco were included as dummy variables as these did not meet the assumption of linearity. The results were highly comparable, with a few small but notable changes. In Model 1, exposure to friends purchasing tobacco “often” was significantly positively associated with impulse purchases compared to “never” (OR = 2.16, 95%CI = 1.14–4.11, *p* = .019). Seeing family purchasing tobacco “often” was no longer significant compared to “never.” In Model 2, smoking accessories were found to be significantly negatively associated with impulse purchases (OR = 0.88, 95%CI = 0.78–0.99, *p* = .040). Exposure to friends purchasing tobacco “often” was significantly positively associated with impulse purchases compared to “never” (OR=2.18, 95%CI = 1.13–4.22, *p* = .021). Seeing family purchasing tobacco “rarely” was no longer significant compared to “never.”

## Discussion

This study aimed to examine the relationship between 6 different sources of tobacco exposure in the retail environment with impulse purchases among adults who smoke. We also examined the influence of neighborhood disadvantage as well as various individual smoking characteristics. Five of the 6 sources of exposure to tobacco in the retail environment were individually associated with impulse purchases: seeing cigarette packages at the counter or checkout, seeing advertisements for tobacco, seeing other people smoking at the entrance of the shop and seeing friends or family buying cigarettes. These findings indicate that several potential sources of exposure could play a role in impulse purchasing of tobacco products. Those seeing family purchasing tobacco (rarely and sometimes compared to never) had the highest odds when accounting for other sources of exposure.

We also investigated the association between tobacco exposure in the retail environment independently of any exposure to POS displays on impulse purchasing, given the well-documented link between POS displays and impulse purchases.^2,8^ In these additional analyses, only respondents who indicated that they never buy tobacco from a specialist shop were included, as POS displays are only permitted in specialist shops in the Netherlands.^30^ These analyses showed that in shops where POS displays and advertising are not permitted, two sources of exposure remained significantly associated with making an impulse purchase, namely seeing advertisements for tobacco, and seeing a family member purchase tobacco. Notably, those who never visit specialty shops to purchase tobacco report seeing tobacco advertising. This may be because some stores do not comply with the legislation and/or that customers, for example, see brand expressions on other products/accessories that they see as advertising. An observational study of 4 Dutch cities found that tobacco visibility (including advertising, product visibility, and external elements such as signboard and posters) was reported in 26.7% of nonexempt shops after the introduction of the POS display ban.^31^ Another possibility is that merely being confronted with the possibility to purchase tobacco products (by seeing it is available or seeing others doing so) is perceived as tobacco advertisement. This notion is supported by findings from Burton et al. ^10,32^ who found that simply seeing a tobacco outlet or tobacco signage can act as a cue for smoking and can thus trigger an impulse purchase and Hoek, Gifford, Pirikahu, Thomson, and Edwards (2010)^6^ in which participants spoke about how the ease of tobacco elicited desire and temptation. These findings provide support for limiting the sale of tobacco to specialist shops only, to reduce the instances of impulse purchasing when shopping for non-tobacco items when with others or by oneself. Such a policy measure could also reduce relapse purchasing for those who are trying to quit smoking and avoid specialty shops.

Greater mean exposure to tobacco was reported by people who were younger in age, lived in a disadvantaged neighborhood, had a greater intention to quit, had previous quit attempt(s) in the past 12 months and a greater frequency of tobacco purchasing. The current study only found one significant interaction between individual characteristics and exposure in the association with impulse purchases, namely, the interaction between exposure and previous quit attempts. One explanation is that those who have recently tried to quit smoking may be more susceptible to impulse purchasing when exposed to tobacco in the retail environment because they may be trying to reduce the amount they are smoking and therefore feel a greater urge to buy when prompted by external exposure. While the other interactions were not found to be significant, other groups who reported greater exposure are at an increased risk of impulse purchases and as a result, reduced likelihood of cessation.^1^ Impulse purchases were individually associated with younger age, making a quit attempt in the past 12 months, intention to quit in the next six months and greater frequency of tobacco purchase, which is consistent with previous literature.^3,4,11^ These findings highlight key groups who could benefit from a reduction in the availability of tobacco products, among those are groups where smoking prevalence is often already higher in the Netherlands, such as younger individuals and those living in a disadvantaged neighborhood.^33,34^ Moreover, outlet density- and/or clustering-based licensing systems could address disparities in exposure between disadvantaged and non-disadvantaged neighborhoods by issuing fewer licenses and/or enforcing minimum distances between locations in disadvantaged neighborhoods.^14,35,36^

This study has a number of strengths and limitations. First, a strength of our design is that we were able to control for several other factors that have previously been shown to be associated with impulse purchases, namely age, past quit attempts, intention to quit, and frequency of tobacco purchase. Second, while previous studies have focused on POS cigarette displays, advertising and promotions, we expanded the sources of exposure to include exposure to tobacco through the actions of others in the retail environment as potential triggers for an impulse purchase. While we measured exposure to friends and family buying tobacco, it is likely that people also see strangers buying tobacco, which may trigger impulse purchasing in the same way that seeing friends and family may. Future research could benefit from including exposure to seeing anyone buying tobacco. Additionally, whilst exposure to tobacco advertising was reported, the nature of that advertising was not explored further, which is particularly relevant for understanding people’s experiences in shops in which advertising is banned. Potentially, there are other retail environment triggers for impulse purchases that we have not measured, such as seeing other nicotine and/or tobacco products or seeing signage for tobacco. It is important to acknowledge that both exposure and impulse purchases may be underreported as the measures are reliant on participant recall.^37^ In particular, impulse purchases are not consciously made^4,8^ and thus participants may not always be able to discern between planned and unplanned purchasing. Finally, impulse purchases are included in the frequency of tobacco purchase. When frequency of purchase was included in the model, this may have clouded the relationship between exposure and impulse purchases, although there were only slight differences between the two models.

## Conclusion

Several sources of exposure to tobacco in the retail environment are associated with impulse purchases of tobacco. Notably, the most influential factors seemed to be seeing cigarettes at the checkout and seeing family members purchase tobacco. Those living in disadvantaged neighborhoods are at an increased risk of exposure to tobacco in the retail environment. These findings provide further support for limiting the sale of tobacco to specialist shops to prevent impulse purchases.

## Supplementary material

Supplementary material is available at *Nicotine and Tobacco Research* online.

ntaf026_suppl_Supplementary_Tables_S1-S2

## Data Availability

The data underlying this article will be shared on reasonable request to the corresponding author.

## Aknowledgments

The authors would like to acknowledge Math Candel for his support with the statistical analyses.

## References

[1] Wood L , Gazey A, Murray K, Kennington K. Unplanned purchasing of tobacco products: Beyond point of sale display. Health Promot J Austr. 2020;31(1):140–144. doi: https://doi.org/10.1002/hpja.260 31102423

[2] Carter OBJ , Mills BW, Donovan RJ. The effect of retail cigarette pack displays on unplanned purchases: results from immediate postpurchase interviews. Tob Control. 2009;18(3):218–221. doi: https://doi.org/10.1136/tc.2008.027870 19264731

[3] Clattenburg EJ , Elf JL, Apelberg BJ. Unplanned cigarette purchases and tobacco point of sale advertising: a potential barrier to smoking cessation. Tob Control. 2013;22(6):376–381. doi: https://doi.org/10.1136/tobaccocontrol-2012-050427 23138525

[4] Wakefield M , Germain D, Henriksen L. The effect of retail cigarette pack displays on impulse purchase. Addiction. 2008;103(2):322–328. doi: https://doi.org/10.1111/j.1360-0443.2007.02062.x 18042190

[5] Slater SJ , Chaloupka FJ, Wakefield M, Johnston LD, O’Malley PM. The impact of retail cigarette marketing practices on youth smoking uptake. Arch Pediatr Adolesc Med. 2007;161(5):440–445. 17485618 10.1001/archpedi.161.5.440

[6] Hoek J , Gifford H, Pirikahu G, Thomson G, Edwards R. How do tobacco retail displays affect cessation attempts? Findings from a qualitative study. Tob Control. 2010;19(4):334–337. doi: https://doi.org/10.1136/tc.2009.031203 20671091

[7] Li L , Borland R, Fong GT, et al Impact of point-of-sale tobacco display bans: findings from the International Tobacco Control Four Country Survey. Health Educ Res. 2013;28(5):898–910. doi: https://doi.org/10.1093/her/cyt058 23640986 PMC3772332

[8] Robertson L , McGee R, Marsh L, Hoek J. A systematic review on the impact of point-of-sale tobacco promotion on smoking. Nicotine Tob Res. 2014;17(1):2–17. doi: https://doi.org/10.1093/ntr/ntu168 25173775 PMC4832971

[9] He Y , Shang C, Huang J, Cheng K-W, Chaloupka FJ. Global evidence on the effect of point-of-sale display bans on smoking prevalence. Tob Control. 2018;27(e2):e98–e104. doi: https://doi.org/10.1136/tobaccocontrol-2017-053996 29332006

[10] Burton S , Spanjaard D, Hoek J. An investigation of tobacco retail outlets as a cue for smoking. Austr Marketing J. 2013;21(4):234–239.

[11] Paul CL , Mee KJ, Judd TM, et al Anywhere, anytime: Retail access to tobacco in New South Wales and its potential impact on consumption and quitting. Soc Sci Med. 2010;71(4):799–806. doi: https://doi.org/10.1016/j.socscimed.2010.05.011 20554363

[12] Siahpush M , Shaikh RA, Robbins R, et al Point-of-sale cigarette marketing and smoking-induced deprivation in smokers: results from a population-based survey. BMC Public Health. 2016;16:302. doi: https://doi.org/10.1186/s12889-016-2992-2 27121197 PMC4848780

[13] Biener L , Albers AB. Young adults: vulnerable new targets of tobacco marketing. Am J Public Health. 2004;94(2):326–330. doi: https://doi.org/10.2105/ajph.94.2.326 14759950 PMC1448251

[14] van Deelen TRD , Veldhuizen EM, van den Putte B, Kunst AE, Kuipers MAG. Socioeconomic differences in tobacco outlet presence, density, and proximity in four cities in the Netherlands. BMC Public Health. 2023;23(1):1515. doi: https://doi.org/10.1186/s12889-023-16347-7 37558979 PMC10413623

[15] Lee JG , Sun DL, Schleicher NM, et al Inequalities in tobacco outlet density by race, ethnicity and socioeconomic status, 2012, USA: results from the ASPiRE Study. J Epidemiol Commun Health. 2017;71(5):487–492. 10.1136/jech-2016-208475 PMC5458784 28249990

[16] Caryl F , Shortt NK, Pearce J, Reid G, Mitchell R. Socioeconomic inequalities in children’s exposure to tobacco retailing based on individual-level GPS data in Scotland. Tob Control. 2020;29(4):tobaccocontrol–tobaccocon2018. doi: https://doi.org/10.1136/tobaccocontrol-2018-054891 PMC7116585 31278083

[17] Marsh L , Doscher C, Cameron C, Robertson L, Petrovic-van der Deen FS. How would the tobacco retail landscape change if tobacco was only sold through liquor stores, petrol stations or pharmacies? Aust N Z J Public Health. 2020;44(1):34–39. doi: https://doi.org/10.1111/1753-6405.12957 31913549

[18] Glasser AM , Onnen N, Craigmile PF, Schwartz E, Roberts ME. Associations between disparities in tobacco retailer density and disparities in tobacco use. Prev Med. 2022;154:106910. doi: https://doi.org/10.1016/j.ypmed.2021.106910 34921833 PMC8750533

[19] Eng L , Su J, Habbous S, et al Tobacco retail availability and tobacco cessation in lung and head and neck (HN) cancer survivors. J Clin Oncol. 2019;37(15_suppl):11559–11559.

[20] Halonen JI , Kivimäki M, Kouvonen A, et al Proximity to a tobacco store and smoking cessation: a cohort study. Tob Control. 2014;23(2):146–151. doi: https://doi.org/10.1136/tobaccocontrol-2012-050726 23436138

[21] Fry R , Burton S, Williams K, et al Retailer licensing and tobacco display compliance: are some retailers more likely to flout regulations? Tob Control. 2017;26(2):181–187. doi: https://doi.org/10.1136/tobaccocontrol-2015-052767 27060100

[22] Netherlands Food and Consumer Product. Uitstalverbod: hoe mag ik de rookwaren presenteren in mijn winkel? https://www.nvwa.nl/onderwerpen/tabak-en-rookwaren-verkopen/reclame-en-uitstalverbod/uitstalverbod. Accessed February 5, 2025.

[23] Ministry of Health Welfare and Sport. *Nationaal Preventieakkoord: Naar een gezonder Nederland*. Ministerie van Volksgezondheid Welzijn en Sport, 2018:76. https://www.rijksoverheid.nl/documenten/convenanten/2018/11/23/nationaal-preventieakkoord. Accessed November 30, 2022

[24] Rijksoverheid. Verbod verkoop tabak supermarkten. https://www.rijksoverheid.nl/actueel/nieuws/2020/11/20/verbod-verkoop-tabak-supermarkten. Accessed May 4, 2023.

[25] Nagelhout GE , Poole NL, Metze M, et al Reducing the number and types of tobacco retail outlets in the Netherlands: Study protocol for a comprehensive mixedmethods policy evaluation. Tob Prev Cessation. 2023;9(March):1–11. doi: https://doi.org/10.18332/tpc/161825 PMC10054766 37009236

[26] Leidelmeijer K , Middeldorp M, Marlet G. LEEFBAARHEID IN NEDERLAND 2018: EEN ANALYSE OP BASIS VAN DE LEEFBAAROMETER 2018. RIGO, Atlas voor gemeenten. https://leefbaarometer.nl/resources/Leefbaarheid+in+Nederland+2018.pdf. Accessed February 5, 2025

[27] Mandemakers J , Leidelmeijer K, Burema F, Halbersma R, Middeldorp M, Veldkamp J. Leefbarometer 3.0: Instrumentontwikkeling.. Atlas Research, In.Fact.Research. https://www.leefbaarometer.nl/resources/LBM3Instrumentontwikkeling.pdf. Accessed April 20, 2023.

[28] Arts K , van Gaalen R, van der Laan J, et al Berekenwijze Sociaal Economische Status scores. Centraal Bureau voor de Statistiek. https://www.cbs.nl/-/media/_pdf/2021/45/berekenwijze-sociaal-economische-statusscores.pdf. Accessed April 20, 2023

[29] Driezen P , Abdullah AS, Quah ACK, Nargis N, Fong GT. Determinants of intentions to quit smoking among adult smokers in Bangladesh: findings from the International Tobacco Control (ITC) Bangladesh wave 2 survey. Global Health Res Pol. 2016;1(1):11. doi: https://doi.org/10.1186/s41256-016-0012-9 PMC5693557 29202060

[30] Nederlandse Voedsel- en Warenautoriteit. Tabaksspeciaalzaak? Registratie en regels. https://www.nvwa.nl/onderwerpen/tabak-en-rookwaren-verkopen/tabaksspeciaalzaak-registratie-en-regels. Accessed February 5, 2025.

[31] Borowiecki M , Deelen TRD, Putte B, Kunst AE, Kuipers MAG. Bans on tobacco display, advertising and vending machines in the Netherlands: impact on visibility of tobacco and compliance of retailers. Tob Control. 2023;34(1):tc-2023-058045. doi: https://doi.org/10.1136/tc-2023-058045 37532434

[32] Burton S , Hoek J, Nesbit P, Khan A. “Smoking is bad, it’s not cool…yet I’m still doing it”: Cues for tobacco consumption in a ‘dark’ market. J Bus Res. 2015;68(10):2067–2074. doi: https://doi.org/10.1016/j.jbusres.2015.03.004

[33] Lenthe FJ , Mackenbach JP. Neighbourhood and individual socioeconomic inequalities in smoking: the role of physical neighbourhood stressors. J Epidemiol Community Health. 2006;60(8):699–705. doi: https://doi.org/10.1136/jech.2005.043851 16840760 PMC2588087

[34] Bommelé J , Willemsen MC. *Kerncijfers roken 2022: de laatste cijfers over roken, stoppen met roken en het gebruik van elektronische sigaretten*. 2023:12. AF2090. 1 February 2023. https://www.trimbos.nl/aanbod/webwinkel/af2090-kerncijfers-roken-2022/

[35] Craigmile PF , Onnen N, Schwartz E, Glasser A, Roberts ME. Evaluating how licensing-law strategies will impact disparities in tobacco retailer density: a simulation in Ohio. Tob Control. 2021;30(e2):e96–e103. doi: https://doi.org/10.1136/tobaccocontrol-2020-055622 32826386 PMC7897331

[36] Luke DA , Hammond RA, Combs T, et al Tobacco town: computational modeling of policy options to reduce tobacco retailer density. Am J Public Health. 2017;107(5):740–746. 28398792 10.2105/AJPH.2017.303685 PMC5388950

[37] Krugman HE. Memory without recall, exposure without perception. J Advertising Res. 2000;40(6):7–14.

