# Supplementary material for: Is Seeing Cigarettes in the Retail Environment Associated With Impulse Purchases? Findings From Surveys in Disadvantaged and Non-disadvantaged Neighborhoods in the Netherlands
Source: Nicotine Tob Res. 2025 Feb 7;27(7):1274–83. doi: 10.1093/ntr/ntaf026 (PMC12187512; doi:10.1093/ntr/ntaf026)
Supplement: ntaf026_suppl_Supplementary_Tables_S1-S2 [file ntaf026_suppl_supplementary_tables_s1-s2.docx]

**Supplementary Table 1** Reported frequency of six sources of tobacco exposure in the shop environment (n=1223) (n, %).

|  | Never | Rarely | Sometimes | Often | Very often |
| --- | --- | --- | --- | --- | --- |
| Cigarette packages at the counter or checkout | 678  (55.4) | 229  (18.7) | 182  (14.9) | 91  (7.4) | 43  (3.5) |
| Smoking accessories (for example lighters, filters, rolling paper) | 324  (26.5) | 195  (15.9) | 342  (28.0) | 266  (21.7) | 96  (7.8) |
| Advertisements for tobacco | 841  (68.8) | 220  (18.0) | 117  (9.6) | 32  (2.6) | 13  (1.1) |
| People smoking at the entrance of the shop | 239  (19.5) | 239  (19.5) | 431  (35.2) | 232  (19.0) | 82  (6.7) |
| A friend buying cigarettes | 613  (50.1) | 23  (18.2) | 276 (22.6) | 84  (6.9) | 27  (2.2) |
| A family member buying cigarettes | 721  (59.0) | 185  (15.1) | 216  (17.7) | 76  (6.2) | 25  (2.0) |

**Supplementary Table 2** Reported frequency of six sources of tobacco exposure in the shop environment, among those who never buy cigarettes in tobacco specialty shops (n=601) (n, %).

|  | Never | Rarely | Sometimes | Often | Very often |
| --- | --- | --- | --- | --- | --- |
| Cigarette packages at the counter or checkout | 353  (58.7) | 105  (17.5) | 77  (12.8) | 40  (6.7) | 26  (3.4) |
| Smoking accessories (for example lighters, filters, rolling paper) | 176  (29.3) | 90  (15.0) | 161  (26.8) | 122  (20.3) | 52  (8.7) |
| Advertisements for tobacco | 448  (74.5) | 94  (15.6) | 42  (7.0) | 12  (2.0) | 5  (0.8) |
| People smoking at the entrance of the shop | 139  (23.1) | 121  (20.1) | 201  (33.4) | 104  (17.3) | 36  (6.0) |
| A friend buying cigarettes | 340  (56.6) | 107  (17.8) | 121  (20.1) | 22  (3.7) | 11  (1.8) |
| A family member buying cigarettes | 384  (63.9) | 88  (14.6) | 94  (15.6) | 25  (4.2) | 10  (1.7) |
